# Latency modulation of collicular neurons induced by electric stimulation of the auditory cortex in *Hipposideros pratti*: *In vivo* intracellular recording

**DOI:** 10.1371/journal.pone.0184097

**Published:** 2017-09-01

**Authors:** Kang Peng, Yu-Jie Peng, Jing Wang, Ming-Jian Yang, Zi-Ying Fu, Jia Tang, Qi-Cai Chen

**Affiliations:** School of Life Sciences and Hubei Key Lab of Genetic Regulation & Integrative Biology, Central China Normal University, Wuhan, China; Universidad de Salamanca, SPAIN

## Abstract

In the auditory pathway, the inferior colliculus (IC) receives and integrates excitatory and inhibitory inputs from the lower auditory nuclei, contralateral IC, and auditory cortex (AC), and then uploads these inputs to the thalamus and cortex. Meanwhile, the AC modulates the sound signal processing of IC neurons, including their latency (i.e., first-spike latency). Excitatory and inhibitory corticofugal projections to the IC may shorten and prolong the latency of IC neurons, respectively. However, the synaptic mechanisms underlying the corticofugal latency modulation of IC neurons remain unclear. Thus, this study probed these mechanisms via *in vivo* intracellular recording and acoustic and focal electric stimulation. The AC latency modulation of IC neurons is possibly mediated by pre-spike depolarization duration, pre-spike hyperpolarization duration, and spike onset time. This study suggests an effective strategy for the timing sequence determination of auditory information uploaded to the thalamus and cortex.

## Introduction

Sensory modalities should be rapid processed for animals to survive. Latency (i.e., first-spike latency) has been thought as a potentially fast, effective, and reliable coding strategy [1–4]. Previous studies showed that latency contains large amounts of sensory stimulus-related information, including sound location [1], touch location and direction [5–6], light contrast [7], and odor identity [8].

In the auditory system, latency plays an important role in signaling sound source location [1] and can be affected by several sound parameters. The latency of most auditory neurons shortens with increasing sound intensity [9] and discharge spikes locked to the onset of sound [10–11]. The latency of onset response varies with the rise time of sound stimulus and generally shortens as the rise time reduces [1,10]. Moreover, latency is tuned to sound frequency, such as having the shortest latency at the center (i.e., best frequency, BF) within the frequency-receptive field (i.e., frequency tuning curve) and the longest latency at its periphery [4,12]. Furthermore, latency depends on specific binaural combinations of sound parameters from two ears because the central auditory neurons receive input from two ears. In particular, the stimulus parameters at the excitatory ear can strongly influence latency. However, the stimulus parameters at the inhibitory ear exert a relatively minimal effect on latency [1,13–14]. Several special central auditory neurons present with “paradoxical latency-shift,” i.e., long response latency to high-intensity sound but short response latency to low-intensity sound; these neurons may play special roles in hearing (e.g., echo-delay processing) [15–16].

Auditory information processing can be explained by neural interactions through the interplay between excitation and inhibition. Sound-driven latency information is transmitted along the ascending pathway only when the projection from one processing stage to the next is highly specific [4]. In reality, aside from synaptic transmission delay, different convergent inputs to central auditory neurons modify the latency in various ways (i.e., “central mechanism”), such as the leading inhibition produces the “paradoxical latency-shift” [15–16]. Sharply tuned intracortical excitation and broadly tuned inhibition shorten and prolong the integration time for spike generation at the BF and off-BF, respectively, to generate latency tuning for sound frequency [4]. Moreover, subcortical auditory signal processing is adjusted and modulated by the corticofugal system. Studies on subcortical auditory nuclei showed that the response latency, frequency, intensity, and spatial domains can be modulated by the corticofugal system via frequency-dependent facilitation and inhibition [17–20]. Excitatory and inhibitory corticofugal feedback input can shorten and prolong the latency of inferior colliculus (IC) neurons, respectively [16,20]. These findings indicate that the central mechanism is crucial in latency modification. However, “peripheral mechanisms” may modify latency in various ways [1,21–25].

The IC is a major center for temporal and spectral integrations under the auditory cortex (AC). It can process and integrate practically all ascending acoustic information from lower auditory centers or nuclei and accept corticofugal modulation from the AC when processing sound signals [26–27]. However, the synaptic mechanisms underlying the modulating latency of IC neurons via the descending pathway from the AC to the IC remain unclear. Therefore, we investigated the synaptic mechanisms underlying the corticofugal latency modulation of IC neurons via the descending pathway from the AC to the IC. The descending facilitation and inhibition from the AC to the IC modulate the latency of IC neurons, and latency modulation is possibly mediated by pre-spike depolarization duration (pre-spike DD), pre-spike hyperpolarization duration (pre-spike HD), and spike onset time (SOT). This study suggests an effective strategy for controlling the sequence determination of auditory information uploaded to the thalamus and cortex.

## Materials and methods

### Ethics statement

All experiments were conducted with the approval of the Institutional Animal Care and Use Committee of Central China Normal University, Wuhan, Hubei, PRC (Permit Number: ccnu2017640-0066). All surgery and recording were performed under sodium pentobarbital anesthesia, and all efforts were made to minimize suffering.

### Experimental preparation

Nine *Hipposideros pratti* (6 males and 3 females; 40–60 g, body weight (b.w.)) obtained from a cave (N:29°26′0.32″; E:114°01′20.49″) near Xianning City, Hubei Province, China were used in this study. The bats were captured using a ground-level mist net. A mist net (2.0 m × 3.0 m) was opened for 6 h from dusk until midnight (mist net was inspected every 10 min). All bats were housed socially in an animal room (dimensions: 3.0 m×3.0 m×3.0 m) and were exposed to local photoperiod and constant temperature (28–30°C) and humidity (>60% rel. humidity). The bats constantly had free access to water and food (mealworm). The bats were examined daily for any sign of weakness, such as empty stomach or slow response to hand-holding. Bats in poor physiological condition were excluded. The Forestry Department of Hubei Province provided permission to conduct the study on this site. No specific permissions were required for our research because *H*. *pratti* is not considered an endangered or protected species. The surgical procedures were basically the same with those in our previous studies [28–29]. In brief, the flat head of a 1.8 cm nail was glued on the exposed skull of each Nembutal-anesthetized bat (45–50 mg/kg b.w.) with acrylic glue and then dentally cemented for 1 or 2 days before the recording session. Exposed tissues were treated with an antibiotic (Neosporin) to prevent inflammation. Each bat was administered with the neuroleptanalgesic Innovar-Vet (Fentanyl 0.04 mg/kg b.w. Droperidol 2 mg/kg b.w.) and then placed inside a bat holder suspended in an elastic sling inside a custom-made double-wall sound-proof room (temperature: 28–30°C).

After fixing the heads of the bats with a set screw, small holes (200–500 μm) were made in the skull above the IC and the AC for orthogonal insertion of glass pipette electrode and custom-made tungsten electrode (see below). Glass pipette electrodes filled with 1 M tri-potassium citrate (impedance: 23–104 MΩ) were used to record the sound-activated responses intracellularly. The glass pipette electrodes were pulled from a single glass barrel (Sutter, USA) with an out-diameter of 1.5 mm, a thick wall, and a thin filament inside the cavity with a microelectrode puller (Sutter, P-97, USA). The recording depth was read using a microdrive scale (SM-21, Narishige, Japan). A common indifferent electrode was placed at the nearby temporal muscles. Additional doses of anesthetics (one fourth of original) were administered during later phases of recording when the animal showed signs of discomfort, such as increase in respiration and minor limb movements. In addition, a local anesthetic (Lidocaine) was applied to the open wound area for reducing any possible pain. Before the recording session, a brief sound was administered to the anesthetized bats for testing their body functional state. Bats are considered to be in good physical condition if they can generate the pinna reflex caused by a given sound and show mild respiratory movements. A bat with good physiological condition was used for three recording sessions on separate days, and each recording session typically lasted 2–6 h (the bat was monitored every 30 min) to minimize the number of animals used. Between recording sessions, the scalp of the bat was treated with antibiotic cream (Neosporin) to prevent inflammation. In addition, the skin was stitched back to the normal position before being placed into a wire mesh cage with a wire bottom (dimensions: 0.8 m ×0.8 m ×0.8 m) of animal room. The bat was then fed with food and water *ad libitum* until the next experimental session. No bats showed signs of illness, and no mortality was observed prior to the experimental endpoint. At the end of the experiment, the bat was sacrificed with an overdose of sodium pentobarbital.

### Acoustic stimulation

For acoustic stimulation, continuous sine waves from a waveform generator (33500B Series, Agilent, USA) were formed into 7 ms pure tone pulses with 0.5 ms rise—decay times and delivered at 2 pulses per second (2 Hz) by a custom-made tone burst generator driven by a stimulator (Master-8, A.M.P.I., Israel). The tone pulses were then amplified after passing a decade attenuator (LAT- 45, Leader, Japan) before they were fed into a small loudspeaker (AKG model CK 50, 1.5 cm in diameter, 1.2 g, frequency response 1–100 kHz). The loudspeaker was placed 30 cm away from the bat’s ear and 30° contralateral to the recording site. Loudspeaker calibration was conducted with a 1/4-inch microphone (4939, B&K, Denmark) placed at the bat’s ear using a measuring amplifier (2610, B&K, Denmark). The loudspeaker output was expressed in decibel sound pressure level (dB SPL) in reference to 20 μPa root mean square. The frequency-response curve of the loudspeaker was plotted to determine the maximal available sound amplitude at each frequency. The maximal stimulus amplitude ranged from 110 dB SPL to 125 dB SPL between 10 and 80 kHz but dropped off sharply to 80 dB SPL at 100 kHz thereafter.

### Electric stimulation of the AC

Electric stimulus signals, which are monophasic square wave pulses generated by an electronic stimulator (Master-8, A.M.P.I., Israel), were prepared into a pulse train (single-pulse duration: 0.1 ms; 50 Hz in frequency, train number: 5–20) [30–31]. Electric stimulus intensity was regulated within 20–50 μA, as required. The output of the electronic stimulator was connected to a stimulation isolator (ISO-Flex, A.M.P.I, Israel), and the output of the stimulus isolator was connected to a pair of custom-made tungsten wire electrodes (FHC Inc., Bowdoin, ME, USA) (tip: <10 μm; intertip distance: ≤100 μm) [32] for stimulating and activating the AC of the bat. The gap between the electric ending and the acoustic stimulus onset was fixed at 5–10 ms, and the paired stimulus was delivered to the bat at 2 Hz.

### Recording procedure

We referred to previous studies [33–34] to determine the location of the AC (i.e., primary auditory cortex, AI) according to the location of a branch of the middle brain artery seen through the skull. Then, a pair of custom-made tungsten electrodes for recording the sound-evoked AC responses and for focal electric stimulation in the AC recording site was driven with a depth of ~750 μm below the brain surface until the layer V of the AC ipsilateral to the IC of the bat by using a microelectrode manipulator (SM-21, Narishige, Japan). Afterward, we measured the BFs for exciting neurons at different locations in the AC with acoustic stimuli [19, 35]. Subsequently, the response of AC neurons to acoustic stimulus was collected and transmitted into a filter (50/60 Hz Noise Eliminator, Hum Bug, Canada) and finally into an amplifier (DAM 80, WPI, USA). The recorded post-stimulus-time-histograms were saved in a PC (Tianqi M4300, Lenovo, China) after A/D conversion. A pair of custom-made tungsten electrodes was used as a stimulating electrode.

For *in vivo* intracellular recording, we used blind penetration for isolating neurons from the IC. Electrode penetrations were conducted vertically through the middle part of the exposed dorsal surface of the IC, and electrode placement was based on the surface landmarks viewed with an operating microscope. Subsequently, the electrode was advanced from the outside of the custom-made double-wall sound-proof room with a microelectrode drive (SM-21, Narishige, Japan). The depth referring to the IC surface of each recorded neuron was obtained from the remote controller of the microelectrode drive. Electrode resistance appeared when the IC surface was reached. When the electrode penetrated an IC neuron, a negative membrane potential (resting potential, RP) was detected, and the action potentials (APs) of IC neurons evoked by 12 repetitive sound presentations were recorded (Axon900A, AXON, USA) (Sample rate = 10 kHz). The sound frequency that elicited the most APs at a given amplitude was defined as the BF, and the amplitude that elicited a 50% probability was defined as the minimal threshold (MT) [29]. Once the AP recording stabilized, the response of IC neurons to the BF and amplitude (MT+20 dB) was recorded immediately before and during the focal cortical stimulation. The *in vivo* intracellular recordings typically lasted approximately 8–15 min (maximum 1 h).

### Data analysis

Means and standard deviations (SDs) of the latency were obtained from the IC neuron response to 12 repetitive BF sound pulses delivered at 20 dB above the MT (MT+20 dB) before and during focal electric stimulation of the AC (AC_ES_). Only the means and SDs based on response probabilities of ≥ 0.15 [10] were considered (*p* < 0.05). For each recorded IC neuron, the standard used for latency change induced by AC_ES_ was the mean value (i.e., mean ± SD) and the *p* value (*p* < 0.05, significant change) of the first spike in response to 12 presentations of a given auditory stimulus. The significant effect of AC_ES_ on the mean latency of each IC neuron was determined by using unpaired *t*-tests to compare the latency of the neuron to 12 repetitive stimulus presentations in the control before AC_ES_ condition with its latency to 12 repetitive stimulus presentations in the presence of AC_ES_ [36]. Subsequently, we divided these IC neurons into three categories based on the significant effect of the response pattern of these latencies on IC neurons: the effect of AC_ES_ on pre-spike DD, pre-spike HD, and SOT.

**Fig 1:**
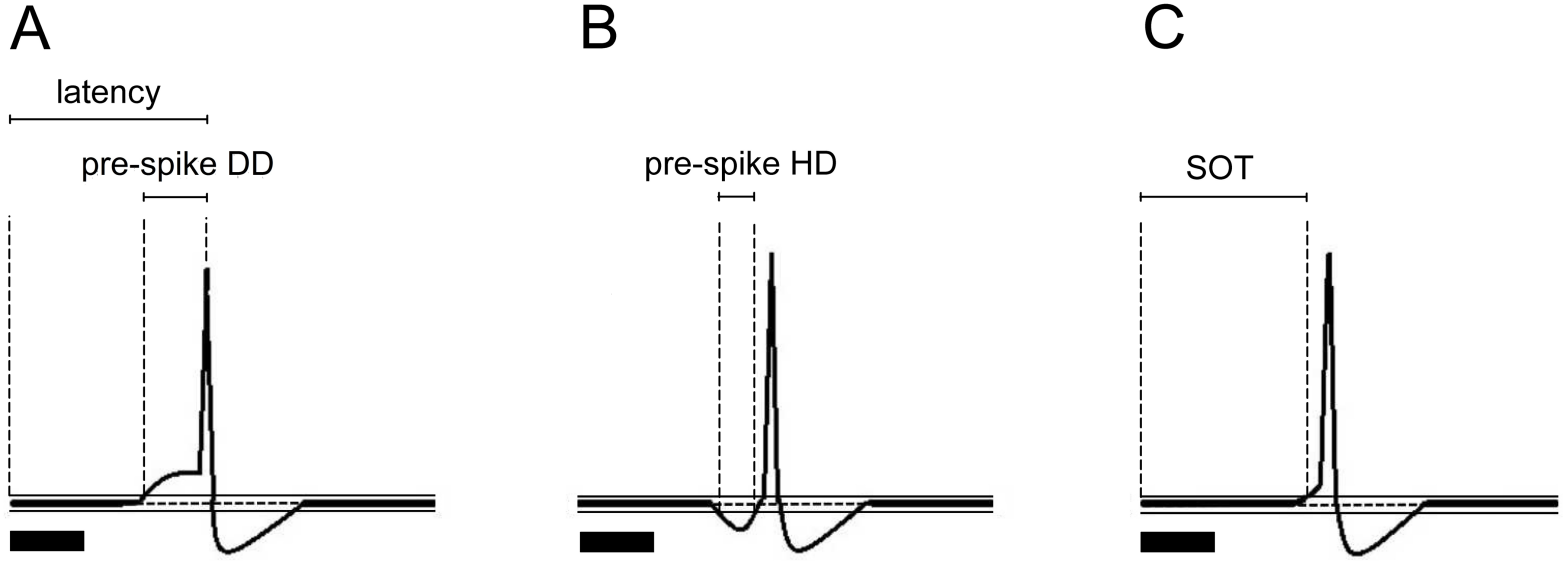
Calculation models of latency, pre-spike DD, pre-spike HD, and SOT. (A) Calculation model of latency and pre-spike DD. (B) Calculation model of pre-spike HD. (C) Calculation model of SOT. Horizontal dashed line on each baseline and two horizontal solid lines above and below the baselines in A, B, and C show the resting potential (RP) and 95% confidence limits. Solid lines on the above left spikes show the pre-spike DD and latency in A, pre-spike HD in B, and SOT in C. Solid black bar under the left side of panels A, B, and C represents the pure tone stimulus.

Depolarization or hyperpolarization was defined as the fluctuations of membrane potentials if they exceeded the baseline by two SDs (95% confidence limits) and occurred within a 50 ms time window after the sound stimulus onset [37]. The latency of IC neurons to sound stimuli was measured from the sound stimulus onset to the peak of the first AP. The pre-spike DD of IC neurons to sound stimuli was measured from the point when depolarization crossed the lines, which defined the confidence limit to the peak of the first AP (Fig 1A) [4]. The pre-spike DD reflected the depolarization rate of the first tone burst evoked AP. The pre-spike HD of IC neurons to sound stimuli was measured from the first point when hyperpolarization crossed the lines, which defined the confidence limit to the second point when hyperpolarization crossed the lines and defined the confidence limit (Fig 1B). The SOT of IC neurons to sound stimuli was measured from the sound stimulus onset to the point when depolarization crossed the lines, which defined the confidence limit (Fig 1C). The SOT reflected the latency of tone-burst-evoked depolarization and the fastest excitatory synaptic input innervating the recorded neuron [4]. Each value of latency, pre-spike DD, pre-spike HD, and SOT of IC neurons in the statistical analyses in the following text was the mean value obtained from the IC neuron response to 12 repetitive BF sound stimulations.

All data obtained in the experiment were processed and plotted using Sigmaplot 10.0. They were quantitatively examined and statistically compared using SPSS 13.0 (Student’s *t*-test at *p < 0*.*05*).

## Results

In this study, 123 neurons (N = 123) were recorded intracellularly from the IC of *H*. *pratti* under pure tone stimulus conditions before and during AC_ES_. The ranges of recording depth, BF, MT, and mean latency of these IC neurons were 2047.88 ± 710.66 μm, 45.04 ± 10.69 kHz, 54.09 ± 16.81 dB SPL, and 13.46 ± 5.44 ms, respectively. According to the effect of AC_ES_ on the latency of IC neurons, the IC neurons (n = 123) obtained in the present study could be classified into three types: facilitation (latency shortening) (n = 20), inhibition (latency prolonging) (n = 23), and unaffected (n = 80). Moreover, three methods (i.e., changing pre-spike DD, pre-spike HD, and SOT) exist in the facilitatory and inhibitory latency modulations activated by AC_ES_.

### Latency change of IC neurons induced by facilitatory modulation activated by focal electric stimulation of the AC

**Fig 2:**
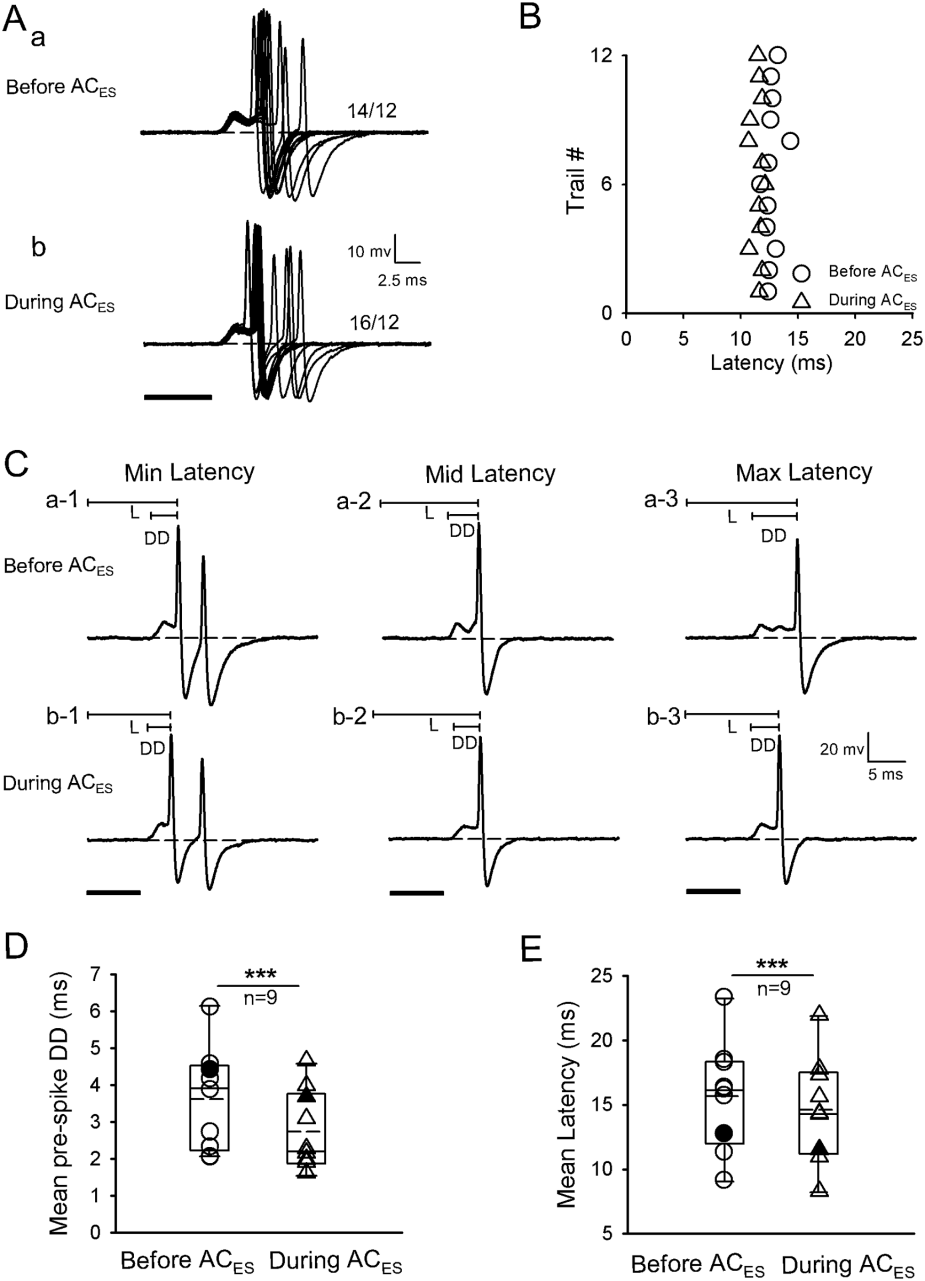
Representative facilitated IC neuron by AC_ES_ showing its latency decreasing induced by pre-spike DD shortening. (A) Original response traces obtained by 12 repetitive pure tone stimulation conditions before (Aa) and during (Ab) AC_ES_. (B) Distribution of latency values calculated according to Aa and Ab before (unfilled circle) and during (unfilled triangle) AC_ES_. (C) Three representative (i.e., minimum (a-1, b-1), middle (a-2, b-2), and maximum (c-1, c-2)) latencies (L) selected from Aa and Ab before (Ca-1–a-3) and during (Cb-1–b-3) AC_ES_. (D) Comparison of mean pre-spike DDs for this type of IC neurons before and during AC_ES_, showing a statistically significant difference (***, *p* < 0.001) between two mean values. The unfilled circle and unfilled triangle show the distribution of pre-spike DD values for this type of IC neurons before and during AC_ES_, respectively. The filled circle and filled triangle show the pre-spike DD value for the representative facilitated IC neuron before and during AC_ES_, respectively. (E) Comparison of mean latencies for this type of IC neurons before and during AC_ES_, showing a statistically significant difference (***, *p* < 0.001) between two mean values. The unfilled circle and unfilled triangle show the distribution of mean latency values for this type of IC neurons before and during AC_ES_, respectively. The filled circle and filled triangle show the latency values of the representative facilitated IC neurons before and during AC_ES_, respectively. Horizontal dashed line on each baseline in A and C shows the RP. The dashed line in the box plots of D and E shows the mean values of pre-spike DD and latency, respectively. The number beside the action potentials (APs) firing in Aa and Ab represents the total number of APs elicited per 12 repetitive trials. Solid lines on the above left spikes show the pre-spike DD and latency in C. Solid black bar under the left side of panels A and C represents the pure tone stimulus. Right angles above the right corners of Ab and Cb-3 show the time and amplitude scales. Vertical bar on top of each column in D and E shows the SD. The recording depth, BF, MT, and mean latency of the representative IC neuron (A–C) are 2501 μm, 45 kHz, 38 dB SPL, and 12.68 ms, respectively.

Three methods (i.e., changing pre-spike DD, pre-spike HD, and SOT) in the facilitatory modulation were activated by AC_ES_ for the latency of IC neurons. The first facilitatory modulation method was shortening the latency of IC neurons by shortening the pre-spike DD during AC_ES_. The latencies of IC neurons (n = 9/120) were shortened with pre-spike DD shortening during AC_ES_. A representative facilitated IC neuron by AC_ES_ is shown in Fig 2. The original response traces obtained by 12 repetitive pure tone stimulations with pre-spike DDs were shorter during AC_ES_ (Fig 2Ab) than before AC_ES_ (Fig 2Aa). Moreover, the scatter plots of latency in Fig 2A show that this IC neuron had a shorter latency during AC_ES_ than before AC_ES_ (Fig 2B). We calculated the pre-spike DDs and three representative (minimum, middle, and maximum) latencies selected from Fig 2A before (Fig 2Ca-1–2Ca-3) and during (Fig 2Cb-1–2Cb-3) AC_ES_, and each latency during AC_ES_ was shorter than the latency before AC_ES_. Statistical analysis showed that the IC neurons had significantly shorter pre-spike DD and latency during AC_ES_ than before AC_ES_ (Fig 2D and 2E) (pre-spike DD: 3.58 ± 1.39 ms (before AC_ES_) vs. 2.71 ± 1.08 ms (during AC_ES_), *p* < 0.001, n = 9, paired *t*-test; latency: 15.66 ± 4.25 ms (before AC_ES_) vs. 14.63 ± 4.10 ms (during AC_ES_), *p* < 0.001, n = 9, paired *t*-test).

**Fig 3:**
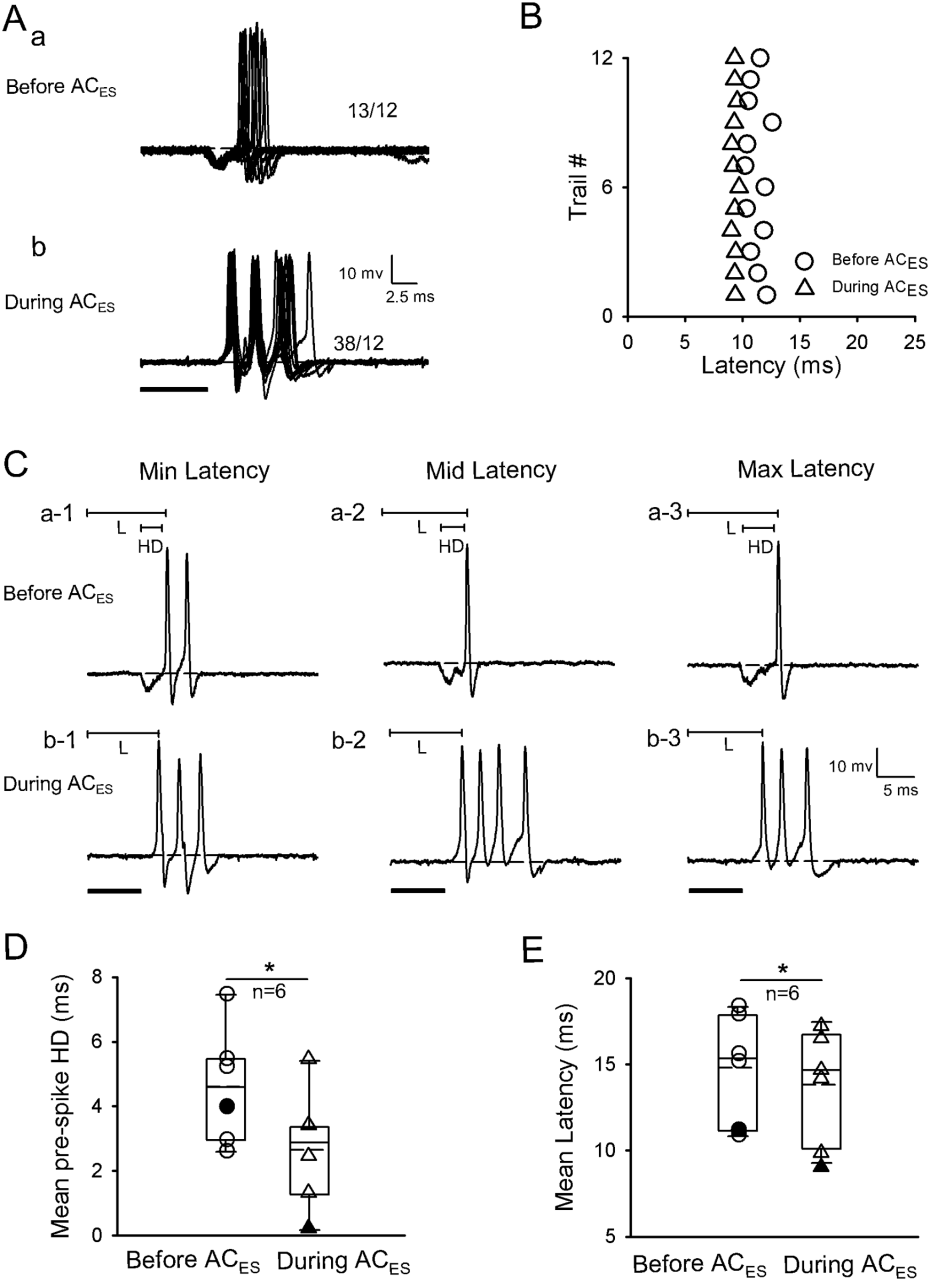
Representative facilitated IC neuron by AC_ES_ showing its latency decreasing induced by pre-spike HD shortening. Original response traces obtained by 12 repetitive pure tone stimulation conditions before (Aa) and during (Ab) AC_ES_. (B) Distribution of latency values calculated according to Aa and Ab before (unfilled circle) and during (unfilled triangle) AC_ES_. (C) Three representative (i.e., minimum (a-1, b-1), middle (a-2, b-2), and maximum (c-1, c-2)) latencies selected from Aa and Ab before (Ca-1–a-3) and during (Cb-1–b-3) AC_ES_. (D) Comparison of mean pre-spike HDs for this type of IC neurons before and during AC_ES_, showing a statistically significant difference (*, *p* < 0.05) between two mean values. The unfilled circle and unfilled triangle show the distribution of pre-spike HD values for this type of IC neurons before and during AC_ES_, respectively. The filled circle and filled triangle show the pre-spike HD value for the representative facilitated IC neuron before and during AC_ES_, respectively. (E) Comparison of mean latencies for this type of IC neurons before and during AC_ES_, showing a statistically significant difference (*, *p* < 0.05) between two mean values. The unfilled circle and unfilled triangle show the distribution of latency values for this type of IC neurons before and during AC_ES_, respectively. The filled circle and filled triangle show the latency value of the representative facilitated IC neuron before and during AC_ES_, respectively. Horizontal dashed line on each baseline in A and C shows the RP. The dashed line in the box plots of D and E shows the mean values of pre-spike HD and latency, respectively. The number beside the APs firing in Aa and Ab represents the total number of APs elicited per 12 repetitive trials. Solid lines on the above left spikes show the pre-spike HD and latency in C. Solid black bar under the left side of panels A and C represents the pure tone stimulus. Right angles above the right corners of Ab and Cb-3 show the time and amplitude scales. Vertical bar on top of each column in D and E shows the SD. The recording depth, BF, MT, and mean latency of the representative IC neuron (A–C) are 2689 μm, 53 kHz, 58.8 dB SPL, and 11.19 ms, respectively.

The second facilitatory modulation method involved latency shortening of IC neurons by shortening pre-spike HD during AC_ES_. In this method, the latencies of six facilitated IC neurons (n = 6/120) were shortened with pre-spike HD shortening induced by AC_ES_. A representative IC neuron is shown in Fig 3. Moreover, the original response traces obtained by 12 repetitive pure tone stimulations with pre-spike HDs were shorter during AC_ES_ (Fig 3Ab) than before AC_ES_ (Fig 3Aa). Furthermore, the scatter plots of latency in Fig 3A show that this IC neuron had a shorter latency during AC_ES_ than before AC_ES_ (Fig 3B). Additionally, we calculated the pre-spike HDs and three representative (minimum, middle, and maximum) latencies selected from Fig 3A before (Fig 3Ca-1–3Ca-3) and during (Fig 3Cb-1–3Cb-3) AC_ES_; each value was shorter during AC_ES_ than before AC_ES_. Statistical analysis showed that the IC neurons had significantly shorter pre-spike HD and latency during AC_ES_ than before AC_ES_ (Fig 3D and 3E) (pre-spike HD: 4.63 ± 1.81 ms (before AC_ES_) vs. 2.69 ± 1.83 ms (during AC_ES_), *p* < 0.05, n = 6, paired *t*-test; latency: 14.86 ± 3.21 ms (before AC_ES_) vs. 13.78 ± 3.40 ms (during AC_ES_), *p* < 0.05, n = 6, paired *t*-test).

**Fig 4:**
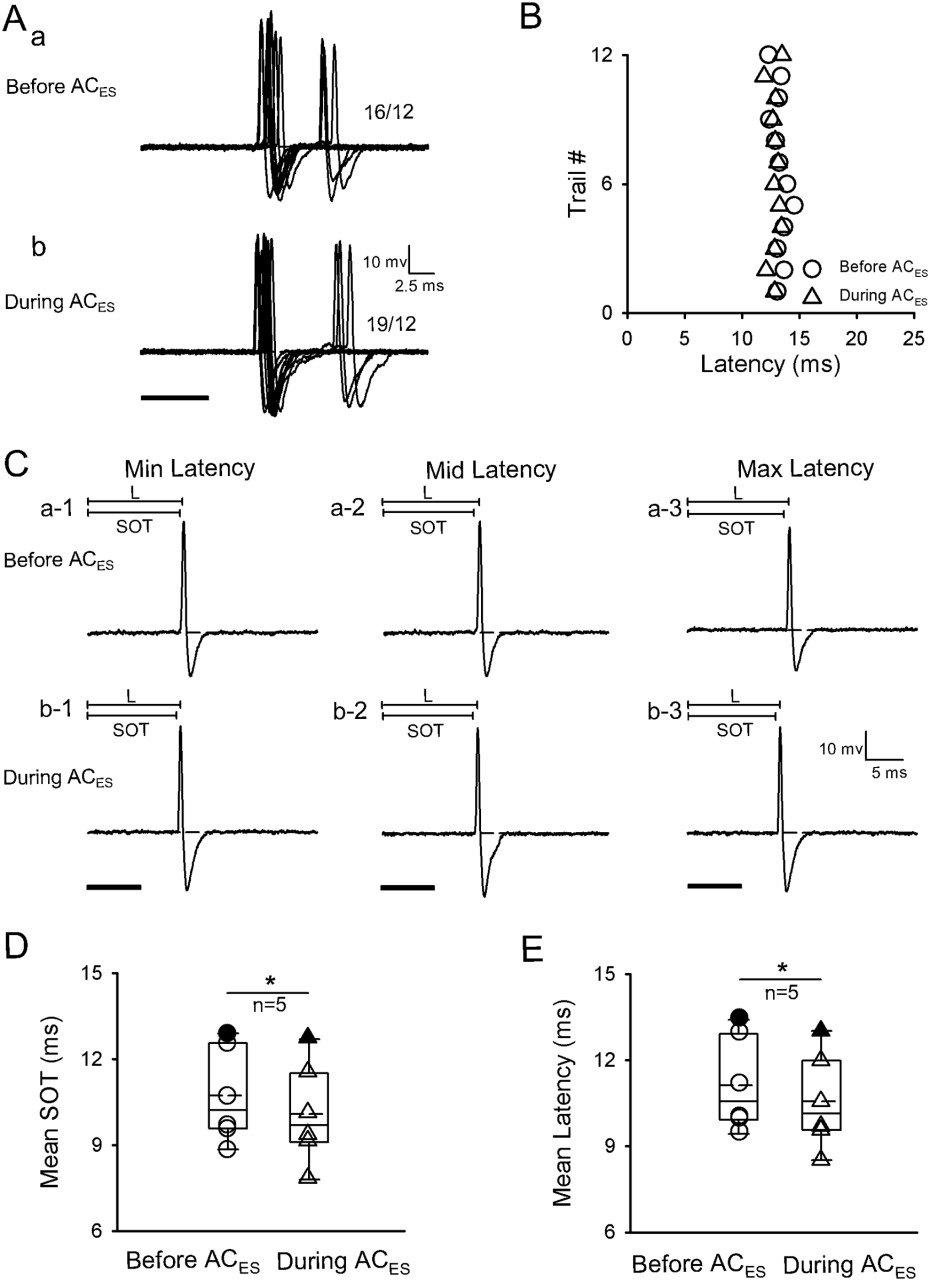
Representative facilitated IC neuron by AC_ES_ showing its latency decreasing induced by SOT forward-shift. Original response traces obtained by 12 repetitive pure tone stimulation conditions before (Aa) and during (Ab) AC_ES_. (B) Distribution of latency values calculated according to Aa and Ab before (unfilled circle) and during (unfilled triangle) AC_ES_. (C) Three representative (i.e., minimum (a-1, b-1), middle (a-2, b-2), and maximum (c-1, c-2)) latencies selected from Aa and Ab before (Ca-1–a-3) and during (Cb-1–b-3) AC_ES_. (D) Comparison of mean SOTs for this type of IC neurons before and during AC_ES_, showing a statistically significant difference (*, *p* < 0.05) between two mean values. The unfilled circle and unfilled triangle show the distribution of SOT values for this type of IC neurons before and during AC_ES_, respectively. The filled circle and filled triangle show the SOT value of the representative facilitated IC neuron before and during AC_ES_, respectively. (E) Comparison of mean latencies for this type of IC neurons before and during AC_ES_, showing a statistically significant difference (*, *p* < 0.05) between two mean values. The unfilled circle and unfilled triangle show the distribution of latency values for this type of IC neurons before and during AC_ES_, respectively. The filled circle and filled triangle show the latency value for the representative facilitated IC neuron before and during AC_ES_, respectively. Horizontal dashed line on each baseline in A and C shows the RP. The dashed line in the box plots of D and E shows the mean values of SOT and latency, respectively. The number beside the APs firing in Aa and Ab represents the total number of APs elicited per 12 repetitive trials. Solid lines on the above left spikes show the latency in C. Solid black bar under the left side of panels A and C represents the pure tone stimulus. Right angles above the right corners of Aa and Cb-3 show the time and amplitude scales. Vertical bar on top of each column in D and E shows the SD. The recording depth, BF, MT, and mean latency of the representative IC neuron (A–C) are 1493 μm, 35 kHz, 34.8 dB SPL, and 13.28 ms, respectively.

The third facilitatory modulation method involved latency shortening of IC neurons through forward shift of their SOT during AC_ES_. In this method, the latencies of five facilitated neurons (n = 5/120) were shortened with forward shift of their SOTs. A representative IC neuron is displayed in Fig 4. The original response traces obtained from 12 repetitive pure tone stimulation conditions from the IC neuron before (Fig 4Aa) and during AC_ES_ (Fig 4Ab) exhibited practically no pre-spike depolarization and pre-spike hyperpolarization. The obtained SOTs and latencies (Fig 4A and 4B) were forward shifted during AC_ES_. Moreover, the scatter plots of latency from Fig 4A show that the IC neuron had a shorter latency during AC_ES_ than before AC_ES_ (Fig 4B). We calculated the SOTs, and three representative (minimum, middle, and maximum) latencies selected from Fig 4A before and during AC_ES_ are shown in Fig 4C. Moreover, earlier SOTs and shorter latencies of IC neurons were observed during AC_ES_ (Fig 4Cb-1–4Cb-3) than before AC_ES_ (Fig 4Ca-1–4Ca-3). Calculation of the SOTs and latencies of these IC neurons before and during AC_ES_ showed that these IC neurons had significantly earlier SOT and shorter latencies during AC_ES_ than before AC_ES_ (Fig 4D and 4E) (SOT: 10.64 ± 1.87 ms (before AC_ES_) vs. 10.08 ± 1.98 ms (during AC_ES_), *p* < 0.05, n = 5, paired *t*-test; latency: 11.00 ± 1.87 ms (before AC_ES_) vs. 10.39 ± 1.87 ms (during AC_ES_), *p* < 0.05, n = 5, paired *t*-test).

### Latency change of IC neurons induced by inhibitory modulation activated by focal electric stimulation of the AC

**Fig 5:**
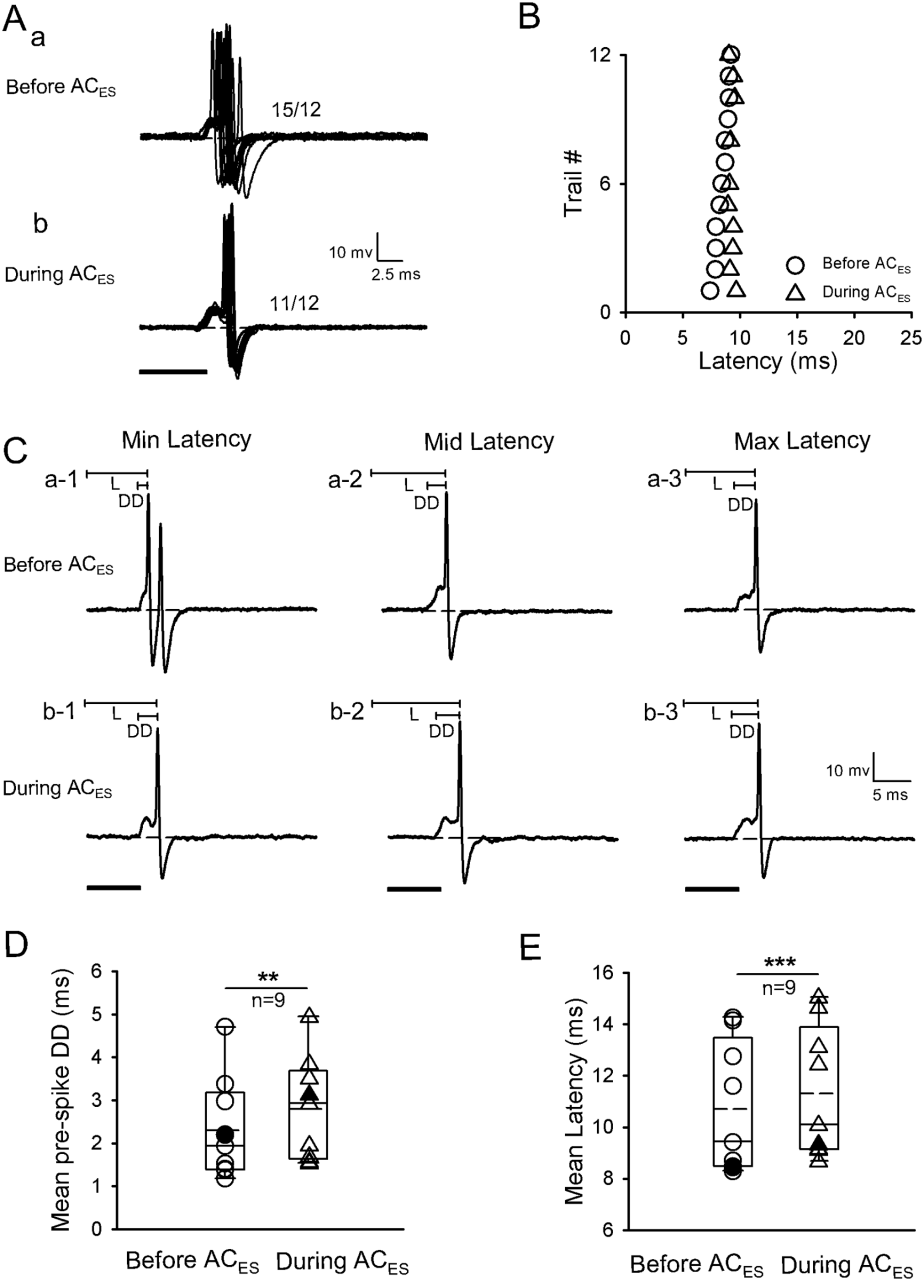
Representative inhibited IC neuron by AC_ES_ showing its latency increasing induced by pre-spike DD lengthening. (A) Original response traces obtained by 12 repetitive pure tone stimulation conditions before (Aa) and during (Ab) AC_ES_. (B) Distribution of latency values calculated according to Aa and Ab before (unfilled circle) and during (unfilled triangle) AC_ES_. (C) Three representative (i.e., minimum (a-1, b-1), middle (a-2, b-2), and maximum (c-1, c-2)) latencies selected from Aa and Ab before (Ca-1–a-3) and during (Cb-1–b-3) AC_ES_. (D) Comparison of mean pre-spike DDs for this type of IC neurons before and during AC_ES_, showing a statistically significant difference (**, *p* < 0.01) between two mean values. The unfilled circle and unfilled triangle show the distribution of pre-spike DD values for this type of IC neurons before and during AC_ES_, respectively. The filled circle and filled triangle show the pre-spike DD value for the representative facilitated IC neuron before and during AC_ES_, respectively. (E) Comparison of mean latencies for this type of IC neurons before and during AC_ES_, showing a statistically significant difference (***, *p* < 0.001) between two mean values. The unfilled circle and unfilled triangle show the distribution of latency values for this type of IC neurons before and during AC_ES_, respectively. The filled circle and filled triangle show the latency value of the representative facilitated IC neuron before and during AC_ES_, respectively. Horizontal dashed line on each baseline in A and C shows the RP. The dashed line in the box plots of D and E shows the mean values of pre-spike DD and latency, respectively. The number beside the APs firing in Aa and Ab represents the total number of APs elicited per 12 repetitive trials. Solid lines on the above left spikes show the pre-spike DD and latency in C. Solid black bar under the left side of panels A and C represents the pure tone stimulus. Right angles above the right corners of Ab and C b-3 show the time and amplitude scales. Vertical bar on top of each column in D and E shows the SD. The recording depth, BF, MT, and mean latency of the representative IC neuron (A–C) are 2574 μm, 59 kHz, 28 dB SPL, and 8.42 ms, respectively.

Three methods (i.e., changing pre-spike DD, pre-spike HD, and SOT) for the inhibitory latency modulation were activated by AC_ES_. The first inhibitory modulation method was prolonging the pre-spike DD of IC neurons in response to pure tone stimulation for prolonging the latency during AC_ES_. Nine (n = 9/120) IC neurons were inhibited by AC_ES_ through this method. A representative inhibited IC neuron is shown in Fig 5. The original response traces obtained by 12 repetitive pure tone stimulations with pre-spike DDs were obviously longer during AC_ES_ (Fig 5Ab) than before AC_ES_ (Fig 5Aa). The scatter plots of latency obtained from Fig 5A show that the IC neurons had longer latencies during AC_ES_ than before AC_ES_ (Fig 5B). Furthermore, we calculated the pre-spike DDs and three representative (minimum, middle, and maximum) latencies selected from Fig 5A before (Fig 5Ca-1–5Ca-3) and during (Fig 5Cb-1–5Cb-3) AC_ES_, and each value during AC_ES_ was longer than before AC_ES_. Statistical analysis showed that the IC neurons had significantly longer pre-spike DD and latency during AC_ES_ than before AC_ES_ (Fig 5D and 5E) (pre-spike DD: 2.18 ± 1.18 ms (before AC_ES_) vs. 2.72 ± 1.19 ms (during AC_ES_), *p* < 0.01, n = 9, paired *t*-test; latency: 10.67 ± 2.52 ms (before AC_ES_) vs. 11.24 ± 2.52 ms (during AC_ES_), *p* < 0.001, n = 9, paired *t*-test).

**Fig 6:**
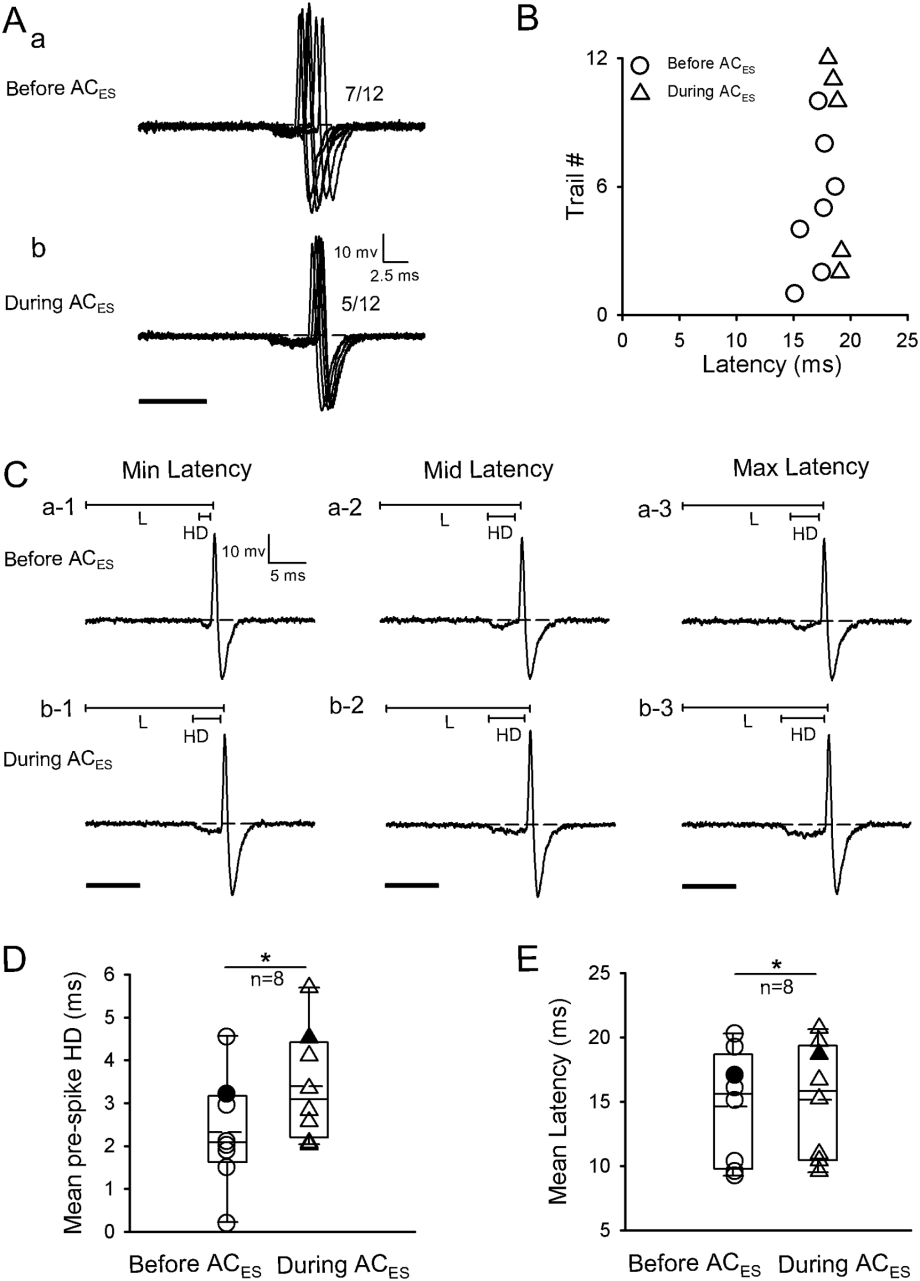
Representative inhibited IC neuron by AC_ES_ showing its latency increasing induced by pre-spike HD lengthening. Original response traces obtained by 12 repetitive pure tone stimulation conditions before (Aa) and during (Ab) AC_ES_. (B) Distribution of latency values calculated according to Aa and Ab before (unfilled circle) and during (unfilled triangle) AC_ES_. (C) Three representative (i.e., minimum (a-1, b-1), middle (a-2, b-2), and maximum (c-1, c-2)) latencies selected from Aa and Ab before (Ca-1–a-3) and during (Cb-1–b-3) AC_ES_. (D) Comparison of mean pre-spike HDs for this type of IC neurons before and during AC_ES_, showing a statistically significant difference (*, *p* < 0.05) between two mean values. The unfilled circle and unfilled triangle show the distribution of latency values for this type of IC neurons before and during AC_ES_, respectively. The filled circle and filled triangle show the latency value of the representative facilitated IC neuron before and during AC_ES_, respectively. (E) Comparison of mean latencies for this type of IC neurons before and during AC_ES_, showing a statistically significant difference (*, *p* < 0.05) between two mean values. The unfilled circle and unfilled triangle show the distribution of latency values for this type of IC neurons before and during AC_ES_, respectively. The filled circle and filled triangle show the latency value of the representative facilitated IC neuron before and during AC_ES_, respectively. Horizontal dashed line on each baseline in A and C shows the RP. The dashed line in the box plots of D and E shows the mean values of pre-spike HD and latency, respectively. The number beside the APs firing in Aa and Ab represents the total number of APs elicited per 12 repetitive trials. Solid lines on the above left spikes show the pre-spike HD and latency in C. Solid black bar under the left side of panels A and C represents the pure tone stimulus. Right angles above the right corners of Ab and C a-1 show the time and amplitude scales. Vertical bar on top of each column in D and E shows the SD. The recording depth, BF, MT, and mean latency of the representative IC neuron (A–C) are 2086 μm, 49.1 kHz, 24.8 dB SPL, and 17.04 ms, respectively.

The second inhibitory modulation method of latency was prolonging the pre-spike HD of IC neurons in response to pure tone stimulation for increasing the latency during AC_ES_. In this method, the latencies of eight inhibited IC neurons (n = 8/120) by AC_ES_ were extended after their pre-spike HDs were prolonged. A representative inhibited IC neuron through this method is displayed in Fig 6. The original response traces obtained under 12 repetitive pure tone stimulations before and during AC_ES_ showed that pre-spike HDs were longer during AC_ES_ (Fig 6A) than before AC_ES_. Meanwhile, the scatter plots of latency from Fig 6A show that the IC neurons had longer latencies during AC_ES_ than before AC_ES_ (Fig 6B). Moreover, we calculated the pre-spike HDs and three representative (minimum, middle, and maximum) latencies selected from Fig 6A showed the longer pre-spike HDs during AC_ES_ (Fig 5Cb-1–5Cb-3) than before AC_ES_ (Fig 6Ca-1–6Ca-3). We calculated the pre-spike HDs and latencies of these IC neurons obtained before and during AC_ES_. Statistical analysis showed that the IC neurons had significantly longer pre-spike HD and latency during AC_ES_ than before AC_ES_ (Fig 6D and 6E) (pre-spike HD: 2.21 ± 1.29 ms (before AC_ES_) vs. 3.30 ± 1.29 ms (during AC_ES_), *p* < 0.05, n = 8, paired *t*-test; L: 14.60 ± 4.38 ms (before AC_ES_) vs. 15.27 ± 4.44 ms (during AC_ES_), *p* < 0.05, n = 8, paired *t*-test).

**Fig 7:**
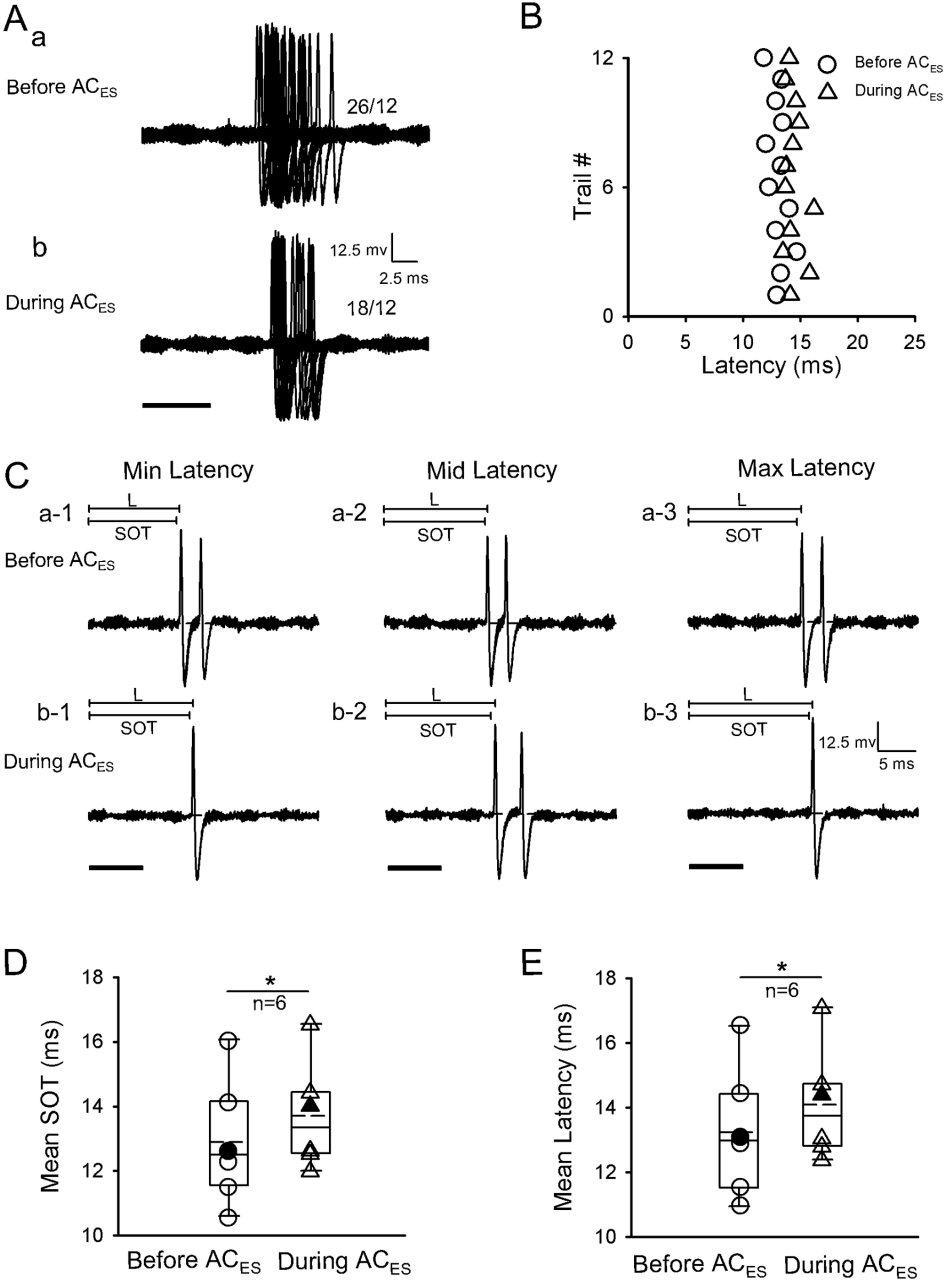
Representative inhibited IC neuron by AC_ES_ showing its latency increasing induced by SOT backward-shift. (A) Original response traces obtained by 12 repetitive pure tone stimulation conditions before (Aa) and during (Ab) AC_ES_. (B) Distribution of latency values calculated according to Aa and Ab before (unfilled circle) and during (unfilled triangle) AC_ES_. (C) Three representative (i.e., minimum (a-1, b-1), middle (a-2, b-2), and maximum (c-1, c-2)) latencies selected from Aa and Ab before (Ca-1–a-3) and during (Cb-1–b-3) AC_ES_. (D) Comparison of mean SOTs for this type of IC neurons before and during AC_ES_, showing a statistically significant difference (*, *p* < 0.05) between two mean values. The unfilled circle and unfilled triangle show the distribution of SOT values for this type of IC neurons before and during AC_ES_, respectively. The filled circle and filled triangle show the SOT value of the representative facilitated IC neuron before and during AC_ES_, respectively. (E) Comparison of mean latencies for this type of IC neurons before and during AC_ES_, showing a statistically significant difference (*, *p* < 0.05) between two mean values. The unfilled circle and unfilled triangle show the distribution of latency values for this type of IC neurons before and during AC_ES_, respectively. The filled circle and filled triangle show the latency value of the representative facilitated IC neuron before and during AC_ES_, respectively. Horizontal dashed line on each baseline in A and C shows the RP. The dashed line in the box plots of D and E shows the mean value of SOT and latency, respectively. The number beside the APs firing in Aa and Ab represents the total number of APs elicited per 12 repetitive trials. Solid lines on the above left spikes show the latency in C. Solid black bar under the left side of panels A and C represents the pure tone stimulus. Right angles above the right corners of Ab and Cb-3 show the time and amplitude scales. Vertical bar on top of each column in D and E shows the SD. The recording depth, BF, MT, and mean latency of the representative IC neuron (A–C) are 3491 μm, 52.6 kHz, 28.5 dB SPL, and 13.07 ms, respectively.

The third inhibitory modulation method for prolonging the latency of IC neurons was to induce a backward shift of SOTs during AC_ES_. The latencies of six IC neurons (n = 6/120) were extended through a backward shift of their SOTs during AC_ES_. A representative IC neuron is displayed in Fig 7. The original response traces obtained from the IC neuron by 12 repetitive pure tone stimulations before (Fig 7Aa) and during AC_ES_ (Fig 7Ab) exhibited practically no pre-spike depolarization and pre-spike hyperpolarization. Comparison analysis showed that the SOTs and latencies (Fig 6A and 6B) of this IC neuron were backward shifted during AC_ES_. Meanwhile, the scatter plots of latency from Fig 7A show that the IC neuron had longer latencies during AC_ES_ than before AC_ES_ (Fig 7B). We calculated the SOTs and three representative (minimum, middle, and maximum) latencies selected from Fig 7A before and during AC_ES_, as shown in Fig 7C. The later SOTs and longer latencies of the IC neuron during AC_ES_ (Fig 7Cb-1–7Cb-3) than before AC_ES_ (Fig 7Ca-1–7Ca-3) were evident. We calculated the SOTs and latencies of IC neurons before and during AC_ES_. The IC neurons had significantly later SOT and longer latency during AC_ES_ than before AC_ES_ (Fig 7D and 7E) (SOT: 12.87 ± 1.96 ms (before AC_ES_) vs. 13.70 ± 1.68 ms (during AC_ES_), *p* < 0.05, n = 6, paired *t*-test; latency: 13.23 ± 2.03 ms (before AC_ES_) vs. 14.06 ± 1.74 ms (during AC_ES_), *p* < 0.05, n = 6, paired *t*-test).

### Relationship between the latency change induced by electric stimulation of the AC and pre-spike DD, pre-spike HD, and SOT of IC neurons

**Fig 8:**
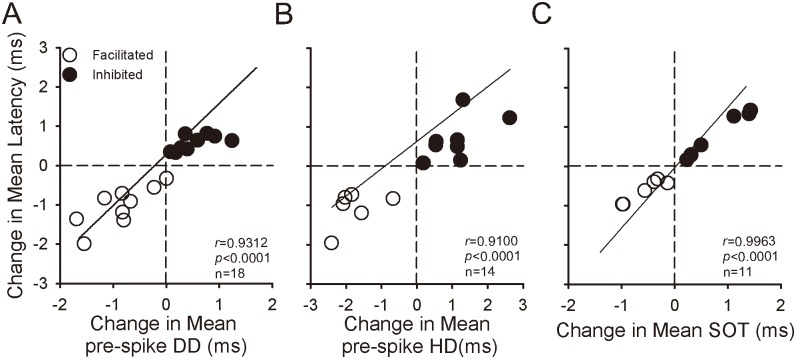
Relation between latency change and pre-spike DD, pre-spike HD, and SOT change of IC neurons. (A) Latency decreasing and increasing with pre-spike DD shortening and lengthening induced by descending facilitation (unfilled circle) and inhibition (filled circle) activated by AC_ES_, respectively (*r* = 0.9312, *p* < 0.0001). (B) Latency decreasing and increasing with pre-spike HD shortening and lengthening induced by descending facilitation (unfilled circle) and inhibition (filled circle) activated by AC_ES_, respectively (*r* = 0.9100, *p* < 0.0001). (C) Latency decreasing with SOT forward shift induced by descending facilitation (unfilled circle) activated by AC_ES_ and vice versa (filled circle) (*r* = 0.9963, *p* < 0.0001). The solid lines in A, B, and C are regression lines.

Scatter plots for the facilitated and inhibited latencies of IC neurons in relation to the changes in pre-spike DD, pre-spike HD, and SOT were prepared to determine if the latency change induced by AC_ES_ is correlated with the changes in pre-spike DD, pre-spike HD, and SOT (Fig 8). Linear regression analyses showed that the latency changes of IC neurons significantly correlated with the changes in their pre-spike DD (Fig 8A), pre-spike HD (Fig 8B), and SOT (Fig 8C) (Fig 8A, *r* = 0.9312, n = 18, *p* < 0.0001; 8B, *r* = 0.9100, n = 14, *p* < 0.0001; 8C, *r* = 0.9963, n = 11, *p* < 0.0001).

### Relationship between the latency change induced by electric AC stimulation and the BF difference between the cortical and collicular neurons

**Fig 9:**
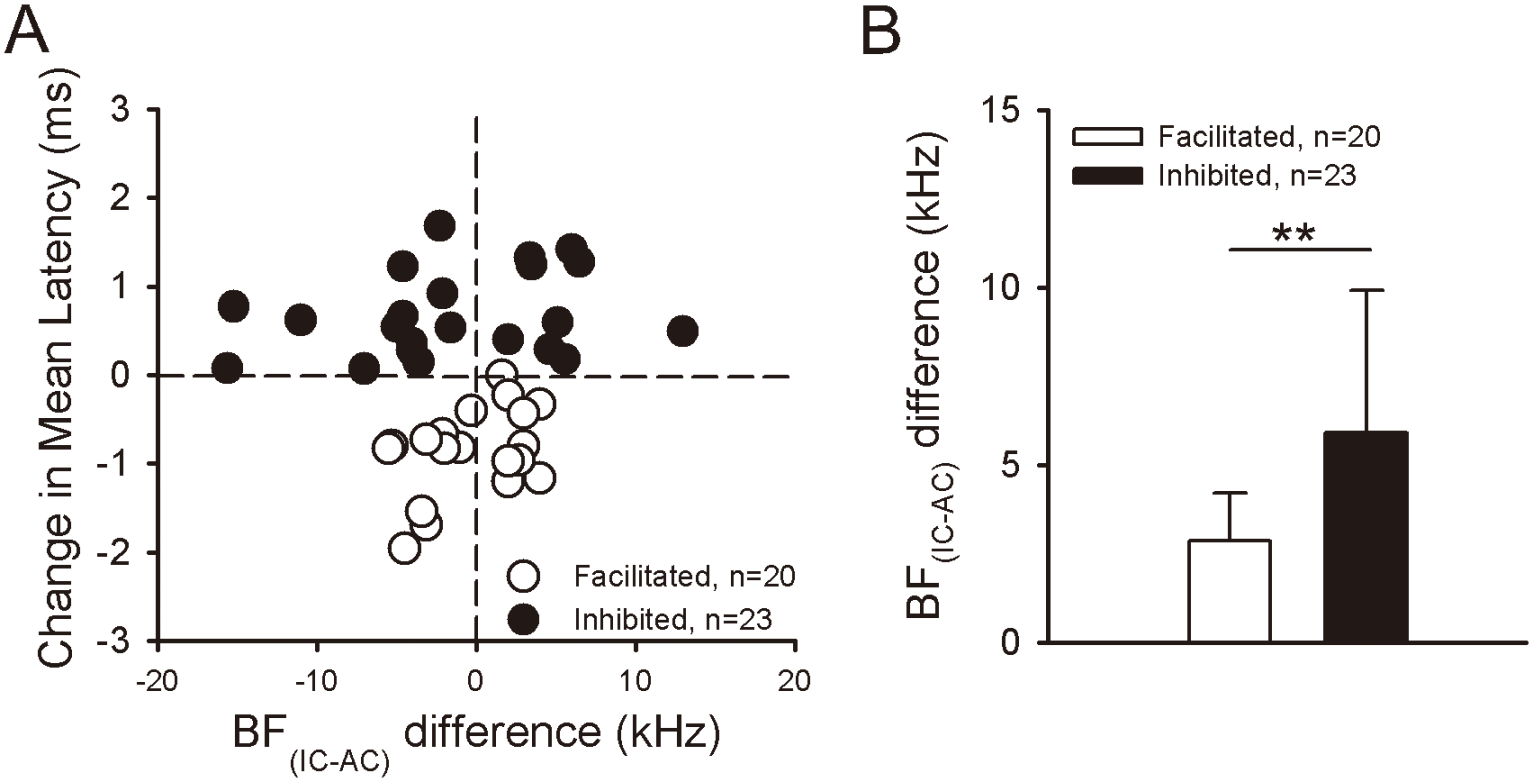
Relation between latency change of IC neurons and the BF_IC-AC_ difference. (A) Scatter plots showing the change in latencies of facilitated (unfilled circle) and inhibited (filled circle) IC neurons against the BF_IC-AC_ difference. (B) Comparison of BF_IC-AC_ difference for corticofugally facilitated (unfilled column) and inhibited (filled column) IC neurons, showing a statistically significant difference (**, *p* < 0.01) between two mean values.

The analysis of 43 IC neurons confirmed that the changes in response latencies evoked by AC_ES_ were related to the BF difference between the cortical and collicular neurons (abbreviated as BF_IC-AC_ difference). The scatter plots show the latency change that resulted from AC_ES_ against the BF_IC-AC_ difference of the corticofugally facilitated and inhibited IC neurons (Fig 9A). We also compared the BF_IC-AC_ difference for the corticofugally facilitated and inhibited IC neurons. The BF difference for corticofugally facilitated IC neurons was smaller than that for corticofugally inhibited IC neurons (Fig 9B, BF_IC-AC_ difference: 2.87 ± 1.33 kHz (corticofugally facilitated IC neurons, n = 21) vs. 5.92 ± 3.99 kHz (corticofugally inhibited IC neurons, n = 23), *p* < 0.05, paired *t*-test;).

## Discussion

The auditory information processing of IC neurons depends on the interplay of different excitatory and inhibitory synaptic inputs to the IC. Moreover, the AC contributes to auditory information processing in IC neurons [18, 37–38]. Previous electrophysiological studies [39–46] found that the corticofugal system can modulate neuron responses (such as spike rate, response latency, and frequency tuning) to multi-sound parameters of sound stimulus in sub-cortical auditory structures. Given that the electric stimulus of the AC is earlier than sound stimulus, the electric activation of the corticofugal system from the AC to the IC basically mimics a corticofugal modulatory effect induced by a prior sound stimulation on the responses of IC neurons evoked by subsequent sound stimulation. In addition, the corticofugal system could be activated by electric stimulation of the AC and inactivated by lidocaine application to the AC [40–43]. The present study demonstrated that the facilitatory modulated IC neurons exhibited a shortened latency of IC neurons (Figs 2, 3 and 4) during AC_ES_. The opposite effects on the latency of IC neurons were observed in the inhibitory modulated IC neurons (Figs 5, 6 and 7). Although these basic modulation effects induced by AC_ES_ are similar to those previously observed [16–17], we found additional modulation details. The results obtained through intracellular recording showed that the AC_ES_-induced changes in the latencies of IC neurons mainly depended on the changes in pre-spike DD (Figs 2, 5 and 8A), pre-spike HD (Figs 3, 6 and 8B), and SOT (Figs 4, 7 and 8C).

The descending fibers from the layer V and even layer VI of the AC terminated in the dorsal nucleus, central nucleus, and external nucleus of the IC [19, 47]. All IC neurons are modulated theoretically by the corticofugal fiber from the AC to the IC. However, AC_ES_ could affect the responses of limited IC neurons that receive fiber projections monosynaptically or polysynaptically from the AC site due to the well-organized topographic projections between the AC and the IC [48–50]. Therefore, in the present study, the corticofugal effect was only observed in 35% (43/120) of IC neurons, which agreed with previous studies [51–52]. The IC neurons were affected by AC_ES_ because AC neurons that transmitted corticofugal fibers to the unaffected IC neurons were not activated by electric stimulation. The site of electric stimulation in the AC is crucial in affecting the responses of IC neurons. A previous study [51] showed that the degree of corticofugal influence on IC neurons decreases with the stimulating electrode moving away from the effective AC stimulation site. In the present study, we first placed the tungsten electrode in the AC and then examined the effect of AC_ES_ on the responses of IC neurons recorded by orthogonally penetrating glass electrode into the IC under an operating microscope. During the experiment, we systematically placed the electrode for electric stimulation at as many places as possible in the AC with the aim of recording many IC neurons affected by AC electric stimulation.

### Effect of electric AC stimulation on the latency of IC neurons

Previous studies showed that corticofugal modulations are based on highly focused positive and widespread negative feedback to subcortical neurons (i.e., frequency-dependent facilitation and inhibition) [16–20, 38–39]. The BF_IC-AC_ difference between corticofugally facilitated and inhibited IC neurons is significant [17]. In the present study, the spike latency change in IC neurons induced by AC_ES_ primarily depended on the relationship between the BF of recorded collicular neurons and the BF of electrically stimulated AC neurons. The BF_IC-AC_ difference for corticofugally facilitated IC neurons is smaller than that for corticofugally inhibited IC neurons (Fig 9). Our results are basically the same with those of a previous study [17].

Neuron spikes are caused by the depolarization of the membrane potential beyond the threshold. A steep depolarization may effectively trigger a neuron to generate spikes. Therefore, the apparent pre-spike depolarization indicates that the excitatory input to the IC neuron may arrive earlier than the inhibitory input. In the present study, the latency change of several IC neurons (n = 18) induced by AC_ES_ varied with the change in pre-spike DD (Figs 2, 5 and 8A). Excitatory and inhibitory corticofugal inputs significantly shortened (Fig 2D, n = 9, *p* < 0.001) and prolonged (Fig 5D, n = 8, *p* < 0.01) the pre-spike DD, respectively. The excitatory corticofugal input to the IC evoked by AC_ES_ overlapping with the ascending excitatory input to the IC evoked by sound stimulus possibly accelerated the rate of sound-evoked depolarization, which caused the IC neurons fire the spike early (Fig 2E, n = 9, *p*<0.001). By contrast, the inhibitory corticofugal input to the IC evoked by AC_ES_ overlapping with the ascending excitatory input to the IC possibly decreased the rate of sound-evoked depolarization, which significantly prolonged the time of IC neurons to fire spikes (Fig 5E, n = 9, *p*<0.001). In the AC, the sharply tuned intracortical excitation and broadly tuned inhibition shortened and prolonged the integration time (i.e., pre-spike DD) for spike generation at the BF and off-BF, which generated latency tuning for sound frequency [4]. These results demonstrate that the corticofugal modulation of the latency through changing pre-spike DD might be the main modulation strategy in the central auditory system. Given that the spike rate of neurons generally reflects the depolarizing current magnitude, the number of spikes increases with increasing excitatory input strength [53]. The present results showed that the spike increased when the neurons were in short latency (Fig 2Aa vs. 2Ab). By contrast, the spike rate of the neurons decreased when the latency was prolonged (Fig 5Aa vs. 5Ab). These results indicate that the response latency and spike rate share a similar mechanism that affects neuron excitability. Latency shortening enhanced the transmission speed and efficiency of auditory information processing. The increase in spike, as a carrier of auditory information, might promote the neural coding and processing of central auditory information. This phenomenon, also termed “one mechanism multiuse,” might widely exist in the central nervous system.

In the present study, other IC neurons (n = 14) showed a pre-spike hyperpolarization that preceded depolarization (Figs 3 and 6) evoked by sound stimulus. Pre-spike hyperpolarization has been documented in several studies [37, 54–56]. The accepted inhibitory inputs by these recorded IC neurons probably arrive slightly earlier than excitatory inputs or simultaneously. The early inhibitory inputs most likely are the convergent inhibitory inputs from the auditory brainstem nuclei below the IC [57–58] or/and from an intrinsic inhibition within the IC [37]. This early inhibition plays an important role in creating the binaural properties of IC neurons [59]. In this study, the excitatory corticofugal input to the IC evoked by AC_ES_ could shorten the sound evoked during pre-spike HD (Fig 3D, n = 6, *p* < 0.05) by partially neutralizing or even almost completely canceling the early inhibition input evoked by sound and eventually shorten the latency (Fig 3E, n = 5, *p* < 0.05) of IC neurons to generate spikes (Fig 3Aa vs. 3Ab). Moreover, the inhibitory corticofugal inputs to the IC evoked by AC_ES_ significantly prolonged the sound evoked by the pre-spike HD (Fig 6D, n = 8, *p* < 0.05) by enhancing the sound evoked during early inhibitory input to the IC, which significantly prolonged the latency (Fig 6E, n = 8, *p* < 0.05) and suppressed the spikes of IC neurons (Fig 6Aa vs. 6Ab). Therefore, the pre-spike hyperpolarization might contribute to form the binaurual properties of IC neurons and adjust the latency by modulating the pre-spike HD by the AC.

In the present study, although the latencies of other IC neuron parts (n = 12) were modulated by AC_ES_ (Figs 4 and 7), they did not exhibit apparent pre-spike depolarization and pre-spike hyperpolarization before and during AC_ES_; they just showed the SOTs to be either significantly shift forward (Fig 4D, n = 5, *p* < 0.05) or significantly backward (Fig 7D, n = 6, *p* < 0.05) during AC_ES_. This phenomenon led to significantly shortened (Fig 4E, n = 5, *p* < 0.05) and prolonged (Fig 7E, n = 6, *p* < 0.05) latencies (Figs 4, 7 and 8C). Previous studies showed that the corticofugal modulation system originated from the AC via descending fibers that project to the medial geniculate body, IC, cochlear nucleus, and superior olivary complex reaching the cochlea through olivocochlear fibers [18,47,60]. Thus, we speculated that the AC_ES_-induced facilitatory and inhibitory latency modulation of latencies of IC neurons without obvious pre-spike depolarization and hyperpolarization occurred at the auditory nuclei below the IC. Furthermore, this part of IC neurons possibly succeeded in modulating the effect of auditory nuclei below the IC via AC_ES_. However, the exact mechanism underlying the SOT shift induced by AC_ES_ needs further study.

Local electric stimulation of the AC has immediate effects on tone responses in the IC. These immediate corticofugal effects disappear within milliseconds or seconds [50, 61] or 30 min after the end of AC_ES_ [17]. Moreover, the long period combination of acoustic and electric stimulation of the AC could induce a continuing plasticity on tone response in the IC. The continuing plasticity lasts for several hours after the end of AC_ES_ [16,18,19]. In the present study, we used real-time *in vivo* intracellular recording to record the response of IC neurons before and during AC_ES_. Considering that the *in vivo* intracellular recording could not be sustained long-term, we did not measure the time course of latency change by AC_ES_. Our study focused on the synaptic potential change of IC neurons induced by the real-time and local electrical stimulation of the AC.

### Effect of other possible factors

Previous studies suggested that the nuclei below the IC are also subject to corticofugal modulation [18, 47, 60]. Yan and Ehret [18] speculated that part of the corticofugally induced changes in the IC could be mediated via corticofugal influences on the cochlea, cochlear nucleus, and/or superior olivary complex. In the present study, the latency change induced by AC_ES_ may also result from corticofugal influences on the subcollicular nuclei. However, whether all or part of the corticofugal effects are due to a direct top-down control of the inferior colliculus or caused by corticofugal influences on the subcollicular nuclei is still unclear [18, 60].

Adaptation, a decline in neural response or firing rate to a long-term stimulus, occurs at many stages of the auditory system, including the IC [62–64]. Neurons showing adaptation in the IC are under the modulation of the AC [65–66]. In the present study, the persisting time of each stimulus (before and during AC_ES_) delivered was very short (only 6 s). In fact, the total time spent for examining the response of IC neurons to various stimuli was within 5 min. On the basis of previous studies [62, 67–70], the repetitive or persistent stimulus used in the present study could elicit adaptability to some degree. For example, Dean et al. [68–69] reported that the rapid adaptation of IC neurons occurs over hundreds of milliseconds, whereas the slow adaptation of IC neurons occurs over seconds. Moreover, Malinina et al. [70] reported that the responses of 90% of IC neurons are completely recovered from poststimulatory adaptation at an interstimulus interval not exceeding 500 ms. Although we did not examine the adaptability of recorded neurons, several degrees of change were induced by adaptability of the neurons (Fig 5). The tendency of neuron response to persistent stimulus basically did not change. We speculated that adaptation affects the response of recoded IC neurons to stimuli, but possibly minor.

### Possible biological significance of the results

In the real world, natural sounds rarely appear in isolation. Valuable auditory information is constantly combined with irrelevant information [71]. The auditory system should prioritize the upload of valuable information to the AC. The IC, as an important integration and relay center along the auditory pathway, receives and processes ascending auditory information and then uploads it to the thalamus and cortex [26–27]. The facilitatory corticofugal modulation shortens the latency of IC neurons and might accelerate the relay speed of optimal information to the thalamus and cortex. Moreover, the inhibitory corticofugal modulation, which prolongs the latency of IC neurons, might decrease the relay speed of off-optimal information to the AC and form the timing sequence of auditory input from the IC to the AC. Furthermore, the different methods of facilitatory and inhibitory corticofugal latency modulation of IC neurons in the present study are possibly the cellular or sub-cellular bases of plastic results obtained via extracellular recording in previous studies [16–18,20].

In the auditory system, latency plays a dominant role in signaling sound source location [1]. Latency coding (as opposed to a spike rate) is more appropriate for processing the short-duration echolocation signals emitted by bats [72]. The IC neurons of big brown bats (*Eptesicus fuscus*) with short latency have short recovery times [73], and this phenomenon can be observed in the IC neurons of awake rabbits for sound localization [74]. Therefore, for echolocating bats, significantly shortening the latency through the excitatory corticofugal input to the IC may significantly affect the capability to follow the high-pulse repetition rate emitted by bats and provide a cellular basis of the physiological modulation for bats to rapidly respond to echo and track prey.

## Abbreviations

AC: auditory cortex
AC_ES_: focal electric stimulation of AC
AP: action potential
BF: best frequency
IC: inferior colliculus
L: latency
MT: minimal threshold
pre-spike DD: pre-spike depolarization duration
pre-spike HD: pre-spike hyperpolarization duration
RP: resting potential
SD: standard deviation
SOT: spike onset time
